# The Mediterranean Diet for Adolescents with Type 1 Diabetes: A Prospective Interventional Study

**DOI:** 10.3390/nu15214577

**Published:** 2023-10-27

**Authors:** Neriya Levran, Noah Levek, Bruria Sher, Elinor Mauda-Yitzhak, Noah Gruber, Arnon Afek, Efrat Monsonego-Ornan, Orit Pinhas-Hamiel

**Affiliations:** 1 Pediatric Endocrine and Diabetes Unit, Chaim Sheba Medical Center, Edmond and Lily Safra Children’s Hospital, Ramat-Gan 5262000, Israel; noah.levek@sheba.health.gov.il (N.L.); elinor.mauda@sheba.health.gov.il (E.M.-Y.); noah.gruber@sheba.health.gov.il (N.G.); 2 National Juvenile Diabetes Center, Maccabi Health Care Services, Ra’anana 4345020, Israel; brurias@gmail.com; 3 The Institute of Biochemistry, Food Science and Nutrition, The Faculty of Agriculture, Food and Environment, The Hebrew University of Jerusalem, Rehovot 5290002, Israel; efrat.mo@mail.huji.ac.il; 4 Division of Nutrition Unit, Chaim Sheba Medical Center, Ramat-Gan 5262000, Israel; 5 Sackler Faculty of Medicine, Tel Aviv University, Tel Aviv 6997801, Israel; arnon.afek@sheba.health.gov.il; 6 General Management, The Chaim Sheba Medical Center, Tel Hashomer, Ramat-Gan 5262000, Israel

**Keywords:** Mediterranean diet (MED), food frequency questionnaire, Israel Mediterranean diet screener (I-MEDAS), time-in-range (TIR), type 1 diabetes, dietary reference intake

## Abstract

The Mediterranean diet (MED) is highly recommended. Medical nutrition therapy is the cornerstone of diabetes treatment. The primary outcome was to evaluate the change in micronutrient intake of youth with type 1 diabetes before and after a 6-month MED intervention; we also assessed adherence and glycemic control. Twenty adolescents, median age 18 years (interquartile range: 15.5–21), median diabetes duration 9 years (7–14), using continuous glucose monitoring devices, received personalized diet regimes based on MED. At 6 months post-intervention, the caloric intake remained unchanged; however, the carbohydrate proportion was lower (*p* = 0.058), and the intakes of some monounsaturated fats increased (*p* = 0.049). Sodium intake exceeded the recommended daily allowance by 250% (*p* = 0.653), before and after the intervention. For blood glucose, the percent TIR (time-in-range, 70–180 mg/dL) improved from 52% (38–60) to 63% (47–71) (*p* = 0.047). The total insulin dose decreased marginally, from 0.76 u/kg (0.64–0.97) to 0.72 u/kg (0.61–0.89) (*p* = 0.067). BMI z-score and waist circumference did not change (*p* = 0.316 and *p* = 0.161, respectively). Diastolic blood pressure percentile decreased from 73% (68–88) to 69% (50–79) (*p* = 0.028), and LDL cholesterol from 114 mg/dL (105–134) to 104 mg/dL (96–124) (*p* = 0.059). The Israeli Mediterranean diet screener score increased, from 8 (7–11) to 13 points (12–14) (*p* < 0.001). The MED-based intervention in youth with type 1 diabetes is feasible and leads to improvement in monounsaturated fat intake, TIR, and diastolic blood pressure. Other parameters show no change (caloric intake, BMI, and HbA1c).

## 1. Introduction

Type 1 diabetes is a chronic autoimmune disorder characterized by the inability to produce insulin and resulting in dysregulated blood glucose levels [1]. Type 1 diabetes is the third most common chronic disease in the adolescent population; those affected in this age group often exhibit poor glycemic control [2,3]. Proper management of type 1 diabetes is crucial for preventing acute and long-term complications. Alongside insulin therapy, medical nutrition therapy is pivotal for achieving optimal glycemic outcomes and body weight; reduced risk of vascular complications, particularly cardiovascular disease (CVD); and improved overall health outcomes [4].

The nutritional recommendations for children and adolescents with type 1 diabetes are based on guidelines for healthy eating in the general population [4]. Among various dietary approaches, the Mediterranean diet (MED) is recommended for both healthy individuals and those with diabetes [5]. Inspired by traditional dietary patterns of countries bordering the Mediterranean Sea, the MED is based on whole grains, legumes, fruits, vegetables, nuts, low-fat dairy products, fish, olive oil, and limited intakes of meat, processed foods, and sweets [6].

Adherence to MED is evaluated according to the KIDMED. A greater KIDMED score signifies adherence to the MED; conversely, a diminished score implies that the child’s dietary habits are not aligned with the MED [7]. The extent to which adolescents with type 1 diabetes adhere to the Mediterranean diet exhibits variability across different studies. For instance, a study conducted in Spain examined the compliance of 97 adolescents with type 1 diabetes and found that approximately 51% of the participants demonstrated favorable adherence to the diet [7]. Conversely, a cross-sectional study involving 700 participants at baseline reported a mere 2.8% rate of good compliance of MED [8].

Eating patterns resembling the MED are likely to be advantageous for long-term health and reducing risks of CVD and the metabolic syndrome [9]. The vast majority of studies that examined the influence of MED on glycemic control were conducted on the adult type 2 diabetes population. Lower levels of glycated hemoglobin (HbA1c) and fasting blood glucose were reported, as well as lower insulin resistance and mortality [10]. For the type 1 diabetes population, the MED has gained considerable attention due to its potential to enhance glycemic control and metabolic health [8,11].

Data on the impact of MED on metabolic control in adolescents with type 1 diabetes are limited. A five-year cross-sectional study of 500 children revealed improvements in blood sugar levels and CVD risk among those with a higher Mediterranean Diet Index (KIDMED) score [8]. A cross-sectional study conducted in Italy on youth with type 1 diabetes assessed the relation between the KIDMED score and glycemic control. While a definitive causal relationship between MED and health outcomes was not established, the researchers concluded that the MED may serve as an effective strategy in the management of type 1 diabetes in young individuals [12]. Another cross-sectional observational study of children with type 1 diabetes in Spain revealed lower levels of HbA1c and higher percent time-in-range (%TIR) of blood glucose among those whose dietary patterns more aligned with the MED [11]. Bona et al. reported improved LDL cholesterol levels following a 6-month intervention in children [13].

The current body of evidence is insufficient to substantiate the utilization of MED as the primary dietary regimen for adolescents with type 1 diabetes. This warrants further research, particularly in the realm of efficacious nutrition therapy interventions. The aim of the present study was to investigate the impact of a MED intervention on micronutrient distribution among adolescents diagnosed with type 1 diabetes. The secondary objectives of the study encompassed assessment of adherence to the prescribed diet and the management of glycemic control.

## 2. Materials and Methods

### 2.1. Participants and Study Design

A prospective clinical intervention was carried out at the Pediatric Endocrinology and Diabetes Unit located inside the Sheba Medical Center. The eligibility criteria for participation in the study consisted of the following: a diagnosis of type 1 diabetes based on the criteria established by the American Diabetes Association, a minimum of one year prior to enrollment; an age between 12 and 21 years; and the utilization of a continuous glucose monitoring device, specifically Dexcom (San Diego, CA, USA), Medtronic (Northridge, CA, USA) or Libre (Alameda, CA, USA) [14]. The exclusion criteria encompassed those who had a medical history of an eating disorder or any other mental disease, either in the participants themselves or in their first-degree family members. The enrollment of eligible study participants occurred subsequently to their interaction during normal visits to the pediatric diabetic clinic. Informed consent was received from participants who were 18 years of age or older, as well as from the parents or legal guardians of participants who were under the age of 18. Informed consent was obtained from adolescents between the ages of 12 and 18. This facilitated a thorough comprehension and consensus of the study’s protocol and the potential hazards. The Helsinki Committee at Sheba Medical Center granted ethics approval and provided oversight for the study (SHEBA-18-5537-OH-CTIL).

### 2.2. Diet Intervention

At the outset, each participant attended a cooking workshop and received a personalized eating regimen based on the MED. Nutritional instructions were provided to participants and, for those under age 18 years, also their parents. Individual diet instructions and support were provided by a dietitian at weeks 1, 2, 4, 7, 10, 12, and 24, for a total of seven face-to-face meetings. During the first 12 weeks, the dietitian called each participant twice for 10- to 15-minute motivational phone calls. Throughout the study, the dietitian was available to the participants

### 2.3. Mediterranean Diet

The MED that was provided was based on the recommendations of Willett and Skerrett [15]. The diet was characterized by moderate-fat intake, rich vegetable and legume intake, low quantities of red meat; and a preference for poultry and fish over beef and lamb. The primary contributors of additional fat intake consisted of approximately 30 to 45 g of olive oil and/or a small quantity of nuts (five to seven nuts <20 g) per day. The planned macronutrient compositions of the diet were 40–50% carbohydrates, 25% protein, and 35% total fat. There was no caloric restriction, but each participant received a weekly meal plan outlining the main meals and snacks (Supplementary Materials)

### 2.4. Assessment of Nutritional Composition and the Mediterranean Diet Screener Score

We used a food frequency questionnaire (FFQ) that was previously used to determine the dietary intake of the Israeli multiethnic population [16]. The FFQ consisted of 116 common Israeli food items, standard portion sizes, and a section for reporting frequency consumption. We assessed the participants’ habitual food consumption at baseline and at 6 months after the intervention.

Total energy intake (kcal) and both macronutrient and micronutrient intake were calculated using Tzameret software (version 5) and the Israeli food and nutrient database [17]. The distributions of macronutrient and micronutrient intakes as percentages of daily energy consumption were also assessed and compared to dietary reference intakes (DRI) [18]. The Israeli Mediterranean diet screener (I-MEDAS) was adapted from the original 14-item MEDAS and modified to apply to the Israeli population [18]. Each item was assigned a score of 1 if it reflected the individual’s food habits, and a score of 0 otherwise. Hence, the I-MEDAS questionnaire, consisting of 17 items, exhibited a scoring range from 0 to 17. The categories were established such as to categorize adherence levels as low, moderate, or high, with corresponding ranges of 0 to 7, 8 to 10, and 11 to 17, as documented in prior publications [18,19]

### 2.5. Medical History and Anthropometric Measurements

Data regarding the age of diabetes onset, diabetes duration, and other medical diagnoses were retrieved from medical records. At each visit, trained and certified staff followed a standard protocol that measured blood pressure, height, weight, and waist circumference. Blood pressure was evaluated according to the “Clinical Practice Guideline for Screening and Management of High Blood Pressure in Children and Adolescents” [20]. Body mass index (BMI) was calculated as weight (kg)/height squared (m^2^). BMI z-score norms were calculated for children aged 12 to 20 years. For older participants, we extrapolated the BMI z-score from the calculated BMI at age 20 years [20]. BMI z-scores were categorized to normal body weight −1.96–1 (percentile 5.0–84.0), overweight 1.0–1.4 (percentile 84.1–93.3), obese 1.5–2 (percentile 93.4–97.7), and morbidly obese >2 (above 97.7 percentile [21].

### 2.6. Insulin and Glycemic Parameters

The percent time-in-range (%TIR) 70–180 mg/dL (3.9–10 mmol/L), as assessed by a continuous glucose monitoring device, was downloaded at each visit with the dietitian. The insulin dosage was expressed by the mean amount of insulin administered per two weeks, normalized by the individual’s weight in kilograms.

### 2.7. Biochemical Parameters

Blood samples were obtained while maintaining metabolic stability, and various measurements were taken, including HbA1c, total cholesterol, LDL-cholesterol, and HDL-cholesterol. The inclusion criteria for this study encompassed the absence of any occurrence of diabetic ketoacidosis in the month preceding the visit, as well as the requirement for participants to undergo testing after a fasting period of at least 12 h. The laboratory findings encompassed measurements of serum C-reactive protein (CRP), blood urea nitrogen (BUN), creatinine, salt, magnesium, calcium, zinc, phosphorus, vitamin B1, vitamin C, and folic acid. The fasting blood samples were collected both at the beginning of the study and after a period of 6 months. These samples were obtained from a vein in the forearm and subsequently analyzed using the Enzyme-Linked Immunosorbent Assay (ELISA) technique at the laboratories of Sheba Medical Center.

### 2.8. Trial Outcomes

Our primary outcome was the nutritional and mineral status after 6 months of MED. Secondary outcomes were glycemic control, body weight, and waist circumference measures at this time point.

### 2.9. Statistical Analysis

In order to detect a large effect size (Cohen d = 0.8), a sample size of 15 participants was determined to be necessary, with a statistical power of 80% and a significance level of 5% [22]. Categorical variables were described using frequencies and percentages. Continuous variables were expressed as medians and interquartile ranges (IQR, 25th; 75th percentiles). Pre- and post-dichotomous dependent variables were evaluated using McNamar’s test. The Wilcoxon test was used to compare continuous variables before and after the 6-month period. Spearman’s correlation coefficient test was used to study associations between continuous variables; >0.36 was considered as a moderate correlation, while r > 0.67 was considered as a high correlation. Continuous variables were compered between categorical variables using the Mann–Whitney test. All the statistical tests were two-sided, and all *p*-values were adjusted by the false discovery rate. *p*-value < 0.05 was considered statistically significant. The statistical analyses were performed with SPSS software (IBM SPSS STATISTIC version 28, IBM Corp., Armonk, NY, USA, 2021).

## 3. Results

### 3.1. Study Group Characteristics

**Figure 1:**
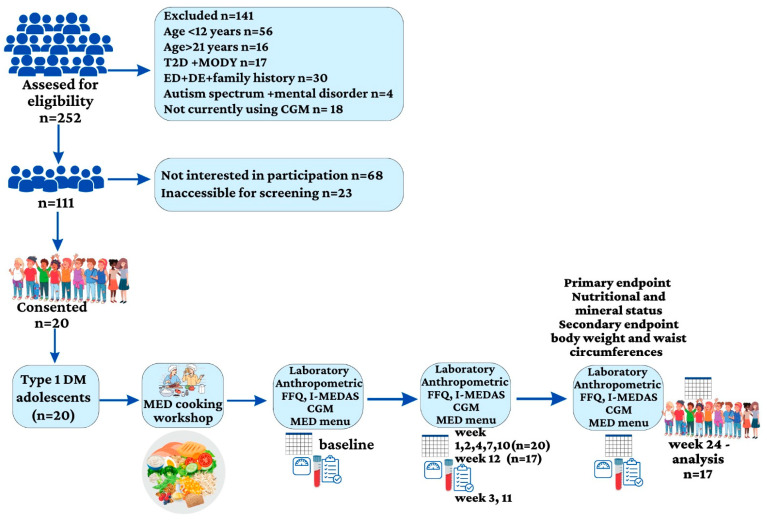
A flow chart of the study. T2D: type 2 diabetes; MODY: maturity-onset diabetes of the young; ED: eating disorder; DE: disordered eating; CGM: continues glucose monitoring; type 1 DM: type 1 diabetes mellitus; MED: Mediterranean diet; FFQ; food frequency questioner; I-MEDAS: Israeli Mediterranean diet screener.

Baseline characteristics of the 20 adolescents (14 females) with type 1 diabetes who were recruited were: a median age (IQR) of 18 years (15.5–21) and a median duration of diabetes of 9 years (7–14). Seventeen participants were treated with insulin pumps and two were on multiple daily injections. The median BMI z-score was 1.1 (0.6–1.3). Nine participants were categorized as having normal weight, eight with overweight and three with obesity. The median waist circumference was 80.0 cm (72.2–90.7); the median waist circumference percentile was 71.0% (35.5–79.0). Three participants dropped out of the study after 12 weeks. One of them was a female who left because she had been on a low-carbohydrate diet prior to the study and was having difficulty adjusting to the amount of carbohydrate intake. Two male participants struggled with the diet framework and had time constraints (Figure 1—A flow chart of the study).

### 3.2. Food Frequency Questionnaire

The median value of total calories at baseline was 2077 kcal (1840–2661): 42% (39–47) of the calories from carbohydrate, 35% (32–37) from fat and 17% (16–20) from protein (Percentages do not add up to 100% because there are other components that contribute to the total daily energy intake, e.g., alcohol. After 6 months of the MED intervention, the percent of calories from carbohydrate decreased to 39% (35–46) (*p* = 0.058) (Table 1).

#### 3.2.1. Median Percentages of Micronutrients According to DRI

The baseline median percentages of iron, potassium, and vitamin E were 75% (59–189), 92% (71–107) and 85% (65–121) lower than the DRIs. Intakes of phosphorus, sodium, copper, vitamin C, riboflavin, pyridoxine, and B12 were more than two-fold higher than the DRIs. After 6 months of the MED intervention, the percentages of DRI (IQR) for iron, potassium, and vitamin E were higher, but with no statistical significance, and still below the recommended DRIs: 84% (60–129), *p* = 0.868; 87% (69–107), *p* = 0.887; and 93% (69–119), *p* = 0.266, respectively. The intakes of phosphorus, sodium, copper, vitamin C, riboflavin, pyridoxine, and B12 remained elevated, and not significantly different from baseline (Table 1).

#### 3.2.2. Median Intakes of Selected Nutrients before and after the MED Intervention

The median percentage of calories derived from ultra-processed foods was 17.7 (13.5–21.8) at baseline, and not significantly different (*p* = 0.255) after the intervention, 15.2 (7.9–21.4) (Table 2). Regarding macronutrients, the intakes of protein and carbohydrates (including fructose, sugar alcohols, and fiber) did not differ significantly between baseline and the end of the 6-month period. While the median total fat intake did not change significantly, the consumption of certain types of fat decreased, and of others increased. Notably, the intakes of cholesterol and saturated fat decreased, while the intakes of monounsaturated fat and polyunsaturated fat showed slight increases. The intake of docosahexaenoic acid (DHA) increased significantly, from 0.101 g (0.0433–0.1614) at baseline to 0.1555 g (0.0474–0.1994) after the intervention (*p* = 0.035). Eicosapentaenoic acid (EPA) intake increased from 0.0266 g (0.0081–0.0419) to 0.0364 g (0.0158–0.0736), *p* = 0.035. Intakes of erucic acid (*p* = 0.031) and docosapentaenoic acid (DPA) (*p* = 0.049) also increased significantly. On the other hand, the intake of parinaric acid decreased significantly (*p* = 0.049). Intakes of all the vitamins and minerals assessed remained stable (Table 2).

### 3.3. I-MEDAS

At the end of the intervention, the median I-MEDAS score was significantly higher, 13 (12–14) points, compared to 8 (7–11) points at baseline (*p* < 0.001). After the 6-month intervention, preferences increased for olive oil (<0.001), and for poultry consumption versus processed meat (*p* = 0.016). Butter and margarine consumption declined (*p* = 0.039), as did the consumption of desserts (*p* = 0.008). Fruit consumption was lower than recommended, and therefore scored zero at baseline and at 6 months (*p* = 0.625). The consumptions of alcohol and salty snacks did not change significantly (Table 3).

### 3.4. Weight Loss, Waist Circumference, and Blood Pressure

The BMI z-score (*p* = 0.316) and waist circumference percentile (*p* = 0.161) did not change significantly (Table 3) during the intervention. At baseline, the median systolic blood pressure was 116 mmHg (109–125) and the diastolic blood pressure was 72 mmHg, (70–79). Both these parameters decreased following the intervention, but the change was statistically significant only for diastolic blood pressure, which decreased to 67 mmHg (71–75), *p* = 0.039. Similarly, blood pressure percentiles followed an identical trend, with a significant change observed in the diastolic percentile (*p* = 0.029) (Table 3).

### 3.5. Glycemic Parameters

Median TIR 70–180 mg/dL increased significantly after the MED intervention, from 52% (38–60) at baseline to 63% (47–71), *p* = 0.047. HbA1c declined from 7.5% (6.8–8.5) to 7.1% (6.7–709), *p* = 0.453. The total amount of insulin decreased from 0.76 U/kg (0.64–0.97) at baseline to 0.72 U/kg (0.61–0.89), *p* = 0.067.

### 3.6. Blood Laboratory Measurements

The median LDL-cholesterol decreased from 114 mg/dL (105–135) at baseline to 104 mg/dL (96–124) after 6 months (*p* = 0.059). Median HDL and triglyceride levels did not change significantly. Blood creatinine (*p* = 0.025), zinc (*p* = 0.031), and potassium levels (*p* = 0.44) were all significantly higher after the intervention. Vitamin C did not differ significantly, before and after the intervention; the respective median values were 11.7 mg/L (9.0–12.9) and 12.0 mg/L (9.0–13.4), *p* = 0.321. However, at baseline, three participants had low levels of vitamin C that increased to the normal range (4.6–14.9 mg/L) after the intervention (Table 4).

### 3.7. Correlations

The I-MEDAS total score was negatively correlated with CRP (r = 0.704; *p* = 0.001). The delta total score was correlated with delta calcium (r = 0.521, *p* = 0.018).

HbA1c was inversely correlated with TIR (r = −0.827; *p* < 0.0001), delta magnesium (r = −0.472; *p* = 0.036), and vitamin C (r = −0.827; *p* = <0.0001). A positive correlation was shown after 6 months between HbA1c and food energy (r = 0.497; *p* = 0.026). TIR was correlated with magnesium (r = 0.470. *p* = 0.037) and vitamin C (r = 0.46; *p* = 0.041), and inversely correlated with delta sugar consumption (r = −0.495; *p* = 0.026).

Body weight and waist circumference were correlated with CRP (r = 0.697; *p* = 0.001 and r = 0.522; *p* = 0.018, respectively). Zinc correlated inversely with CRP (R = −0.453; *p* = 0.045) and with BMI z scores (r = −0.458; *p* = 0.042). Correlations between fiber intake and polyunsaturated fat are shown in Table 5.

Systolic blood pressure was correlated with sodium (r = 0.582, *p* = 0.007). Other correlations are shown in Table 5.

## 4. Discussion

We assessed, before and 6 months after a MED intervention, the intakes and blood levels of macronutrients and micronutrients, the glycemic control, and the anthropometric measurements of adolescents with type 1 diabetes. At baseline, the intakes of sodium and several trace elements were elevated compared to recommended values. We demonstrated that at 6 months, the MED-based intervention was feasible, and that glycemic outcomes and several nutritional parameters were improved.

The participants in our trial were instructed to consume extra-virgin olive oil and nuts on a daily basis. While the daily percentage of calories derived from fat changed only slightly, the composition of fatty acids, particularly docosahexaenoic acid (DHA) and eicosapentaenoic acid (EPA), increased. Associations have been reported between the consumption of nuts and extra-virgin olive oil, which are abundant in unsaturated fatty acids [23] and rich in antioxidants, with improved lipid profiles [24]. While the overall lipid profile did not change significantly during the intervention, 11 participants had lower LDL cholesterol levels after the intervention.

The median %TIR significantly improved after the intervention, as did HbA1C levels. Total sugar consumption correlated inversely with TIR. Our findings corroborate a cross-sectional analysis that involved 97 children with type 1 diabetes, which demonstrated improved HbA1c levels among those with optimal adherence to MED [12]. Another research investigation demonstrated a correlation between higher levels of adherence to the MED and improved glycemic regulation [8]. Although the amount of carbohydrates in grams did not differ significantly before and after intervention, the proportion of carbohydrates in total calories was lower after the diet. This change might have contributed to the observed improvement in glycemic control, as seen in other studies [25]. The intensive nutritional monitoring program, conducted by a registered dietitian, might also have contributed to better glycemic control [26,27].

The median intake of dietary fiber observed in our study met 100% of the DRIs at baseline and 111% after 6 months. Significant positive changes in the consumption of whole grains, nuts, and legumes were evident based on the I-MEADS. All the aforementioned factors contributed to the overall status of fiber intake. Fruit consumption remained unchanged during the intervention period, as indicated by a score of zero at both time points. This suggests lower intake than recommended. Fruits possess substantial concentrations of sugars, and their consumption may impact blood glucose regulation. Thus, individuals with type 1 diabetes often restrict their fruit consumption. A meta-analysis of individuals with type 2 diabetes revealed that the intake of fruits significantly reduced fasting blood glucose levels but did not affect HbA1c concentrations [28]. Fresh fruits are abundant sources of dietary fiber, organic acids, minerals, and antioxidants such as vitamins and polyphenols. One prominent biological process associated with fruit consumption is antioxidation, wherein the conversion of free radicals into more stable forms enhances their scavenging capacity. This process also mitigates the generation of reactive oxygen species by restraining mitochondrial oxidative stress, thereby preserving cellular redox homeostasis [29]. Individuals with type 1 diabetes and their caregivers should be guided regarding appropriate fruit consumption and insulin administration.

A key principle of the MED involves reducing the consumption of highly processed food products. Nevertheless, we report no statistically significant difference in the median proportion of calories derived from processed foods prior to and following the intervention. This finding may be attributed to the limitations of the FFQ to distinguish between enhanced nutritional qualities of food items. For instance, both whole wheat bread and white bread are classified under the same category of ultra-processed foods, despite the former having superior nutritional qualities. Notably, the majority of participants transitioned to this particular type of bread. The use of the I-MEDAS enhanced accuracy and enabled the discernment of important changes in laboratory measures that were associated with distinct food items of the MED.

The intake of the micronutrient sodium is affected by the consumption of the foods mentioned in the previous paragraph. Both at baseline and at 6 months after the intervention, the median sodium intake of our participants was twice that of the recommended daily amount. We also report a significant association between systolic blood pressure and sodium intake. Our findings are consistent with the US SEARCH study in which none of the 190 children and adolescents with type 1 diabetes met the recommended sodium intake [27]. A systematic review and meta-analysis involving 58,531 children and adolescents showed a dose-response relation, such that each additional gram of sodium consumed per day raised systolic and diastolic blood pressure by about 1 mm Hg [30]. Excessive sodium consumption is a major risk factor for high blood pressure and cardiovascular disease [31]. Several studies have reported high sodium intake with high adherence to the MED [32,33]. Hence, reducing the consumption of high-sodium foods deserves specific consideration. The main sources of sodium from the MED might be pickled vegetables, whole wheat breads and canned fish. Indeed, according to the I-MEDAS, fish consumption (especially canned tuna fish) and whole wheat bread were significantly higher after the intervention.

Our data demonstrate a significant increase in plasma zinc levels after 6 months of following the MED. Foods such as fish, eggs, dairy products, beans, nuts, and whole grains are rich sources of zinc [34]. The I-MEDAS results indicate a notable increase in the consumption of fish portions throughout the intervention. This could account for the observed rise in zinc levels. Zinc has been found to potentially enhance glucose uptake into cells by modulating the insulin-stimulated movement of GLUT4 [35]. Zinc is also involved in insulin signaling and the redox signaling pathway, which has been suggested as a mechanism that may explain the association between zinc and cardiometabolic risk [35,36]. Notably, in the current study, plasma zinc levels were negatively correlated with HbA1c and CRP, further emphasizing the importance of the MED.

Dietary copper intake among our participants was two-fold higher than the DRI, but below 20% of the upper intake levels, both before and after the intervention [37]. The existing research on copper abnormalities and diabetes predominantly centers on type 2 diabetes and its association with insulin resistance [38]. Research carried out in the United States reported elevated levels of serum copper in individuals with type 1 diabetes compared with a control group [39]. In addition to elevated glucose levels that initiate free radical reactions, the dysregulation of copper also significantly contributes to the occurrence of oxidative stress, thereby increasing the risk of diabetes complications [39,40]. The primary sources of copper in the MED include seeds and nuts, wheat-bran cereals, whole-grain products, and chocolate. The question arises as to whether more attention is needed regarding excessive copper consumption among individuals with type 1 diabetes.

For our cohort, median serum Mg levels fell within the normal low range and exhibited no significant differences pre- and post-dietary intervention, despite exceeding the DRI for this micronutrient. Notably, serum levels reflect only a fraction of the total Mg present in the body [34]. We report correlations of serum Mg level with Hba1c and TIR, two measures of glycemic control, thus corroborating prior studies [41]. Mg is a vital mineral that functions as a cofactor in more than 300 enzyme systems that have crucial roles in various biochemical reactions, including the regulation of blood glucose and blood pressure [28]. Foods that are rich in dietary fibers and abundant in the MED typically serve as sources of Mg, including green leafy vegetables, such as spinach, legumes, nuts, seeds, and whole grains [34].

No significant differences were observed in BMI z-score and waist circumference following the 6-month intervention. These findings align with the constant calorie intake throughout the intervention. Given that our study did not involve caloric restriction and did not focus on weight loss, we did not anticipate significant changes in anthropometric measures. Body weight and waist circumference did correlate positively with CRP levels. This finding corresponds with a non-randomized descriptive study, in which children diagnosed with type 1 diabetes were selected and matched with a control group according to sex and age [42]. Children with obesity were included in that study. Statistically significant correlations were observed of high-sensitivity CRP with both BMI and waist circumference Z-score [42].

There are several inherent limitations to our study. FFQ interviews are susceptible to recall bias. Nevertheless, this recall method is considered more reliable than a single day recall [43]. Although the FFQ has a robust research foundation, it is likely to be limited in its accurate capture of precise nutrient quantities. Furthermore, the relatively small cohort, only 20 participants, may not provide a comprehensive understanding of the potential nutritional and metabolic effect of the MED. Another limitation is the lack of data on serum levels of several minerals that might have elucidated additional associations between nutrient intakes and their serum levels. Additionally, we did not collect data on polyphenol intake, which is an important component of the MED that has been associated with many health advantages. Furthermore, the absence of information on participants’ physical activity levels is another limitation, as it can play a crucial role in the assessment of metabolic outcomes. On the positive side, the prospective follow-up design is a strength of the study. This work represents a pioneering effort in implementing a 6-month intervention, characterized by rigorous dietary surveillance focused on the MED, and comprehensive evaluation of multiple micronutrients.

In the contemporary context of prevailing exclusionary dietary practices, the MED is distinguished by its emphasis on the inclusion of specific food groups such as fruits, vegetables, whole grains, legumes, nuts, low-fat milk products, and olive oil, which are all recommended for daily consumption. The incorporation of a diverse range of foods without limitations into an individual’s dietary regimen has a dualistic nature. On one hand, it facilitates adherence to the prescribed diet; while on the other hand, the absence of absolute restrictions creates a gray area that permits the consumption of foods that are not deemed advisable. The higher and improved average I-MEDAS score observed after a 6-month period indicates greater adherence to the MED, though several micronutrients and nutritional parameters did not change significantly.

## 5. Conclusions

This 6-month Mediterranean diet (MED) intervention for adolescents with Type 1 diabetes revealed feasibility and improvements in glycemic outcomes and nutritional parameters. Caregivers and healthcare providers should remain vigilant about specific concerns within the MED framework, such as sodium consumption, akin to the DASH diet’s focus. It’s essential to monitor sodium intake and promote alternatives like incorporating herbs and spices for flavor while reducing sodium. Monitoring copper intake and serum levels is important to prevent overconsumption. Encouraging fruit consumption, with proper insulin guidance, is advisable. The ultimate goal is to establish a comprehensive, balanced dietary plan that supports overall well-being and efficient diabetes management. Further studies with larger sample sizes and extended observation periods are needed to validate these findings and enhance dietary guidelines. In summary, the MED shows potential as a viable dietary strategy for managing glycemic levels and improving nutritional status in individuals with Type 1 diabetes.

## Figures and Tables

**Table 1:** Percentages of macronutrients in the diet and median (interquartile range) percentages of micronutrients compared to DRI values.

	Baseline	After 6 Months	Delta	*p*-Value
%Protein from calories	17 (16–20)	18 (16–20)	0.0 (−1.1–2.3)	0.552
%Fat from calories	35 (32–37)	38 (34–40)	2 (−1–5)	0.092
%Carbohydrate from calories	42 (39–46)	39 (35–46)	−2.7 (−8.3–1.5)	0.058
Percent DRI				
Fiber	100 (72–113)	111 (77–133)	0.5 (−8.7–47.2)	0.501
Calcium	120 (75–160)	131 (76–149)	1.5 (−25.7–16)	0.816
Iron	75 (59–189)	84 (60–129)	0.0 (−35–15)	0.868
Magnesium	143 (94–189)	161 (116–195)	21 (0–43)	0.068
Phosphorus	241 (140–271)	221 (149–278)	0 (−27–31)	0.981
Potassium	92 (71–107)	87 (69–107)	−0.5 (−8.5–16.7)	0.887
Sodium	250 (194–332)	251 (186–326)	0.5 (−37.0–25.7)	0.653
Zinc	142 (115–159)	131 (109–167)	0 (−8–20)	0.795
Copper	189 (145–262)	180 (144–245)	0 (−13–49)	0.756
Vitamin C	302 (225–405)	283 (217–358)	1 (−107–111)	0.984
Thiamin B1	127 (110–169)	130 (114–166)	0 (−4–29)	0.408
Riboflavin B2	217 (161–261)	225 (167–266)	0 (−23–56)	0.463
Niacin B3	164 (122–211)	168 (135–224)	5 (−11–42)	0.309
Folate B9	105 (79–137)	105 (86–122)	0 (−17–32)	0.981
Pyridoxine B6	205 (154(259)	200 (160–273)	0 (−30–30)	0.868
Vitamin A	134 (88–196)	131 (110–166)	0 (−17–41)	0.687
Vitamin E	85 (60–121)	93 (69–119)	8 (−9–25)	0.266
Vitamin D	141 (98–246)	210 (83–254)	17 (−3–51)	0.103

The data are presented as medians and interquartile ranges. The percentages of the DRIs were calculated according to the recommended amounts for age and sex. All the data are presented as percentages except the calories. DRI: dietary recommended intake.

**Table 2:** Median (interquartile range) intakes of selected nutrients before and after the Mediterranean diet intervention, according to the Food Frequency Questionnaire.

	Baseline	After 6 Months	Delta	*p*-Value
Total calories, kcal	2077.8 (1840.7–2661.9)	2050.9 (1770.4–2827.5)	0.0 (−306.3–268.3)	0.943
Energy percent ultra-process	17.7 (13.5–21.8)	15.2 (7.9–21.4)	0.0 (−9.4–1.3)	0.255
Protein, g	97.1 (81.7–120.9)	97.9 (79.1–125.8)	0.7 (−11.3–8.2)	0.906
Fat, g	85.7 (71.8–104.8)	89.6 (69.5–104.8)	1.1 (−8.7–15.7)	0.554
Carbohydrate, g	222.0 (188.8–306.0)	197.8 (159.6–278.3)	0.0 (−63.4–25.3)	0.356
Total sugars, g	100.9 (64.1–136.6)	88.7 (70.9–106.9)	−0.2 (−18.4–12.1)	0.523
Sugar alcohols, g	0.28 (0.1–0.7)	0.2 (0.1–1.7)	0.0 (−0.2–0.2)	0.975
Fructose, g	19.4 (11.1–25.5)	19.2 (12.4–25.0)	0.0 (−5.4–4.0)	0.653
Fiber, g	27.3 (19.9–41.3)	28.5 (23.5–42.1)	0.0 (−2.2–8.1)	0.795
Calcium, mg	1283.1 (775.2–1639.8)	1308.4 (899.1–1692.1)	31.9 (−234.7–204.3)	0.554
Iron, mg	11.9 (10.3–17.9)	11.9 (10.4–17.6)	0.0 (−3.1–2.3)	0.981
Magnesium, mg	479.2 (327.9–631.4)	552.8 (398.3–691.4)	66.0 (0.0–128.8)	0.084
Phosphorus, mg	1768.9 (1335.6–2038.1)	1724.4 (1410.9–2023.7)	0.00 (−144.6–356.1)	0.586
Potassium, mg	4304.0 (3338.1–5069.6)	4110.3 (3248.1–5044.1)	−13.7 (−414.7–789.2)	0.906
Sodium, mg	3757.9 (2914.5–4994.4)	3771.1 (2800.2–4900.8)	−2.7 (−551.4–387.2)	0.687
Zinc, mg	12.1 (9.9–14.1)	11.9 (8.8–15.6)	0.0 (−1.4–1.7)	0.906
Copper, mg	1.9 (1.4–2.6)	1.9 (1.4–2.5)	0.0 (−0.1–0.4)	0.723
Selenium, mcg	132.9 (118.4–181.1)	148.9 (105.0–183.0)	0.0 (−17.2–26.7)	0.687
Choline, mg	503.2 (345.7–682.0)	517.6 (339.1–717.9)	0.0 (−145.4–107.3)	0.906
Vitamin A, mcg	2081.0 (1281.1–2597.6)	1934.1 (1202.8–2304.4)	5.19 (−406.6–233.9)	0.723
Vitamin C, mg	228.4 (162.9–304.1)	212.7 (148.1–274.1)	0.0 (−32.8–33.7)	0.981
Thiamin B1, mg	1.3 (1.1–1.9)	1.4 (1.2–1.8)	0.0 (−0.4–0.2)	0.687
Riboflavin B2, mg	2.2 (1.7–3.3)	2.5 (2.0–3.0)	0.0 (−0.3–0.5)	0.653
Niacin B3, mg	23.0 (18.8–29.8)	25.1 (18.4–32.8)	0.0 (−2.3–6.1)	0.523
Vitamin B6, mg	2.6 (1.9–3.3)	2.5 (1.9–3.3)	0.0 (−0.7–0.4)	0.831
Folate B9, mcg	422.3 (316.3–551.2)	423.4 (331.6–488.6)	0.0 (−127.8–127.5)	0.943
Vitamin B12 mcg	5.8 (3.8–7.0)	6.1 (4.1–7.2)	0.0 (−0.8–0.5)	0.981
Vitamin D, mcg	7.0 (4.8–12.3)	9.8 (4.1–12.6)	0.5 (−1.2–2.6)	0.246
Vitamin K, mcg	224.5 (184.3–354.4)	218 (155.6–317.0)	−4.0 (−56.5–35.5)	0.356
Vitamin E, mcg	12.7 (9.0–17.1)	13.2 (10.3–17.2)	0.0 (−3.6–3.7)	0.460
Cholesterol, mg	395.1 (262.6–559.3)	346.0 (242.3–617.1)	0.0 (−99.2–45.4)	0.831
Saturated fat, mg	27.9 (20.7–31.6)	27.1 (19.7–30.9)	−1.1 (−3.1–0.7)	0.287
Monounsaturated fat, g	34.1 (27.0–42.4)	39.3 (29.7–43.2)	2.3 (0.0–11.4)	0.149
Polyunsaturated fat, g	17.7 (15.0–24.2)	18.2 (13.1–25.4)	0.0 (−3.3–3.4)	0.906
Docosahexaenoic acid (DHA), g	0.1018 (0.0433–0.1614)	0.1555 (0.0474–0.1994)	0.0184 (0.0785)	0.035
Palmitoleic acid, g	1.0639 (0.8908–1.3945)	1.0873 (0.7696–1.6299)	−0.0069 (−0.1928–0.2098)	0.943
Parinaric acid, g	0.0196 (0.004–0.0278)	0.0198 (0.0052–0.0556)	−0.0454 (−0.0832; −0.0056)	0.049
Eicosapentaenoic acid (EPA), g	0.0266 (0.0081–0.0419)	0.0364 (0.0158–0.0736)	0.0048 (0.0000–0.0323)	0.035
Erucic acid, g	0.0431 (0.0051–0.0604)	0.0435 (0.0134–0.1145)	0.0042 (0.0000–0.0526)	0.031
Docosapentaenoic acid (DPA), g	0.0220 (0.0117–0.0392)	0.0312 (0.0137–0.0476)	0.0015 (−0.0001–0.0174)	0.049

The data are presented as medians and interquartile ranges. kcal: kilocalories; g: gram; mg: milligrams; mcg: microgram.

**Table 3:** I-MEDAS Israeli Mediterranean diet screener.

		Baseline	Six Months	*p*-Value
Total Score		8 (7–11)	13 (12–14) Delta: 5 (2–6)	<0.001
	The criterion for a positive score			
Preference for olive oil	Yes	Y = 1 (5%) N = 19 (95%)	Y = 18 (90%) N = 2 (90%)	<0.001
Poultry more than red/processed meat	Yes	Y = 12 (60%) N = 8 (40%)	Y = 19 (95%) N = 1 (5%)	0.016
Non-starchy vegetables	2+ servings/d	Y = 16 (80%) N = 4 (20%)	Y = 19 (95%) N = 1 (5%)	0.25
Fruits- without juice	3+ servings/d	Y = 4 (20%) N = 16 (80%)	Y = 6 (30%) N = 14 (70%)	0.625
Butter/Margarine	<1 serving/d	Y = 9 (45%) N = 11 (55%)	Y = 16 (80%) N = 4 (20%)	0.039
Sweet soft drinks	<1 serving/d	Y = 13 (65%) N = 7 (35%)	Y = 18 (90%) N = 2 (10%)	0.063
Whole grains	3 + servings/d	Y = 7 (35%) N = 13 (65%)	Y = 13 (65%) N = 7 (35%)	0.07
Red and ultra-processed meat	<7 servings/wk	Y = 15 (75%) N = 5 (25%)	Y = 15 (75%) N = 5 (25%)	*
Alcohol	7+ servings/wk	Y = 20 (100%) N = 0 (0%)	Y = 20 (100%) N = 0 (0%)	*
Non sweetened dairy	2 + servings/d	Y = 16 (80%) N = 4 (20%)	Y = 17 (85%) N = 3 (15%)	0.999
Legumes	3+ servings/d	Y = 1 (5%) N = 19 (95%)	Y = 8 (40%) N = 12 (60%)	0.016
Fish (fresh& preserved)	3 + servings/wk	Y = 10 (55%) N = 10 (50%)	Y = 17 (85%) N = 3 (15%)	0.016
Nuts	3 + servings/wk	Y = 3 (15%) N = 17 (85%)	Y = 12 (60%) N = 8 (40%)	0.004
Hummus/tahini salad	3 + servings/wk	Y = 7 (35%) N = 13 (65%)	Y = 6 (30%) N = 14 (70%)	0.999
Desserts	<3 servings/wk	Y = 7 (35%) N = 13 (65%)	Y = 15 (75%) N = 5 (25%)	0.008
Savory pastries	≤2 servings/wk	Y = 16 (80%) N = 4 (20%)	Y = 19 (95%) N = 1 (5%)	0.375
Salty snacks	≤3 servings/wk	Y = 16 (80%) N = 4 (20%)	Y = 16 (80%) N = 4 (20%)	*

Total score is presented as medians and interquartile. Significant differences shown in bold; wk: week; d: day; Y = yes; N = no. * The discordant pairs are equal or less than 25, so a chi value was not calculated.

**Table 4:** Clinical and biochemical data of the participants before and after the MED intervention.

		Before	After	Change	*p*-Value
Anthropometric measurements	BMI z-score	1.1 (0.6–1.3)	1.2 (0.5–1.5)	0.0 (−0.1–0.1)	0.316
	Waist circumference percentile	71.0 (35.5–79.0)	59.5 (26.5–77.0)	−1.0 (−5.5–1.7)	0.161
Glucose variables	TIR 70–180 mg/dL	52 (38–60)	63 (47–71)	7 (−1–14)	0.047
	Total daily dose unit/kg	0.76 (0.64–0.97)	0.72 (0.61–0.89)	−0.04 (−0.13–0.00)	0.067
Blood pressure percentiles	Systolic %	64 (38–94)	60 (30–72)	−1 (−28–14)	0.349
	Diastolic%	73 (68–88)	69 (50–79)	−8 (−21–5)	0.028
Blood tests	HbA1c %	7.5 (6.8–8.5)	7.1 (6.7–7.9)	0.0 (−0.6–0.3)	0.453
	Cholesterol mg/dL	176 (160–186)	171 (156–189)	0 (−22–7)	0.293
	LDL cholesterol mg/dL	114 (105–134)	104 (96–124)	−3.5 (−24–1.5)	0.059
	HDL cholesterol mg/dL	64 (58–69)	62 (50–74)	−2 (−8–1)	0.195
	Triglyceride mg/dL	68 (61–95)	72 (51–87)	0 (−16–9)	0.877
	CRP <0.20–5.00 mg/L	1.9 (1.0–5.3)	2.1 (0.5–6.9)	0.0 (−0.3–0.6)	0.744
	Urea 17–45 mg/dL	28.0 (22.2–33.7)	26.0 (20.0–33.7)	0.0 (−7.0–2.0)	0.307
	Creatinine 0.62–1.10 mg/dL	0.70 (0.57–0.79)	0.73 (0.54–0.85)	0.01 (−0.01–0.08)	0.025
	Zinc 50.0–150.0 mcg/dL	119.0 (96.5–140.0)	131.5 (110.5–150.7)	8.5 (0.0–30.7)	0.031
	Calcium 8.1–10.4 mg/dL	9.8 (0.6–10.0)	9.8 (9.5–10.1)	0.0 (−0.2–0.2)	1.000
	Phosphorus 2.00–4.00 mg/dL	4.2 (3.4–4.3)	4.1 (3.5–4.3)	−0.05 (−0.37–0.20)	0.477
	Potassium 3.5–5.1 mmol/L	4.2 (4.1–4.4)	4.4 (4.2–4.6)	0.1 (−0.1–0.4)	0.044
	Magnesium 1.90–2.70 mg/dL	1.9 (1.8–2.0)	1.9 (1.8–2.0)	0.0 (0.0–0.1)	0.424
	Vitamin C 4.6–14.9 mg/L	11.7 (9.0–12.9)	12.0 (9.0–13.4)	0.2 (−0.8–0.9)	0.321
	Vitamin B1 66.5–200.0 nmol/L	144.8 (121.0–171.0)	141.5 (118.3–158.0)	−0.2 (−11.4–4.8)	0.472
	Folic acid 5.9–24.0 ng/mL	8.7 (6.7–12.3)	10.5 (6.2–13.5)	0.0 (−2.3–3.9)	0.679

The data are presented as medians and interquartile ranges. BMI: body mass index; HbA1c: glycated hemoglobin; mg/dL; milligram per deciliter; mg/L: milligrams per liter; mcg/dL: microgram per deciliter; nmol/L nanogram per liter; ng/mL: nanogram per milliliter; TIR, time-in-range; CRP, C-reactive protein.

**Table 5:** Correlations of food energy and various nutrients with delta total daily fiber intake (g).

	R	*p*-Value
Delta food energy	−0.473	0.035
Delta energy from ultra-processed	0.741	<0.0001
Delta total fat	0.566	0.009
Delta carbohydrates	0.659	0.002
Delta calcium	0.771	<0.0001
Delta zinc	0.641	0.002
Delta copper	0.78	<0.0001
Delta vitamin C	0.576	0.008
Delta thiamin	0.891	<0.001
Correlation with delta polyunsaturated fat (g)		
Delta energy from ultra-processed food	0.7	0.001
Delta carbohydrate	0.893	<0.0001
Delta total fiber	0.765	<0.0001
Delta fructose	0.671	0.001
Delta magnesium	0.805	<0.0001
Delta zinc	0.63	0.003
Delta copper	0.784	<0.0001
Delta folate	0.768	<0.0001
Delta thiamine	0.712	<0.0001
Delta vitamin C	0.637	0.003

Delta: after minus before; g: gram.

## Data Availability

The authors can share data upon request.

## Supplementary Materials

The following supporting information can be downloaded at: https://www.mdpi.com/article/10.3390/nu15214577/s1. The following supporting information can be obtained from: neriya.levran@sheba.health.gov.il. This Supplementary S1 has been provided by the authors to provide readers with additional information about this work. Neriya Levran, Orit Pinhas-Hamiel, et al.

## References

[1] DiMeglio L.A., Evans-Molina C., Oram R.A. (2018). Type 1 Diabetes. Lancet.

[2] Wood J.R., Miller K.M., Maahs D.M., Beck R.W., DiMeglio L.A., Libman I.M., Quinn M., Tamborlane W.V., Woerner S.E., T1D Exchange Clinic Network (2013). Most Youth with Type 1 Diabetes in the T1D Exchange Clinic Registry Do Not Meet American Diabetes Association or International Society for Pediatric and Adolescent Diabetes Clinical Guidelines. Diabetes Care.

[3] Khadilkar A., Oza C. (2022). Glycaemic Control in Youth and Young Adults: Challenges and Solutions. Diabetes Metab. Syndr. Obes..

[4] Annan S.F., Higgins L.A., Jelleryd E., Hannon T., Rose S., Salis S., Baptista J., Chinchilla P., Marcovecchio M.L. (2022). ISPAD Clinical Practice Consensus Guidelines 2022: Nutritional Management in Children and Adolescents with Diabetes. Pediatr. Diabetes.

[5] Boucher J.L. (2017). Mediterranean Eating Pattern. Diabetes Spectr..

[6] Mańkiewicz-Żurawska I., Jarosz-Chobot P. (2019). Nutrition of Children and Adolescents with Type 1 Diabetes in the Recommendations of the Mediterranean Diet. Pediatr. Endocrinol. Diabetes Metab..

[7] Serra-Majem L., Ribas L., Ngo J., Ortega R.M., García A., Pérez-Rodrigo C., Aranceta J. (2004). Food, Youth and the Mediterranean Diet in Spain. Development of KIDMED, Mediterranean Diet Quality Index in Children and Adolescents. Public Health Nutr..

[8] Zhong V.W., Lamichhane A.P., Crandell J.L., Couch S.C., Liese A.D., The N.S., Tzeel B.A., Dabelea D., Lawrence J.M., Marcovina S.M. (2016). Association of Adherence to a Mediterranean Diet with Glycemic Control and Cardiovascular Risk Factors in Youth with Type I Diabetes: The SEARCH Nutrition Ancillary Study. Eur. J. Clin. Nutr..

[9] Guasch-Ferré M., Willett W.C. (2021). The Mediterranean Diet and Health: A Comprehensive Overview. J. Intern. Med..

[10] Martín-Peláez S., Fito M., Castaner O. (2020). Mediterranean Diet Effects on Type 2 Diabetes Prevention, Disease Progression, and Related Mechanisms. A Review. Nutrients.

[11] Dominguez-Riscart J., Buero-Fernandez N., Garcia-Zarzuela A., Morales-Perez C., Garcia-Ojanguren A., Lechuga-Sancho A.M. (2022). Adherence to Mediterranean Diet Is Associated with Better Glycemic Control in Children with Type 1 Diabetes: A Cross-Sectional Study. Front. Nutr..

[12] Antoniotti V., Spadaccini D., Ricotti R., Carrera D., Savastio S., Goncalves Correia F.P., Caputo M., Pozzi E., Bellone S., Rabbone I. (2022). Adherence to the Mediterranean Diet Is Associated with Better Metabolic Features in Youths with Type 1 Diabetes. Nutrients.

[13] Cadario F., Prodam F., Pasqualicchio S., Bellone S., Bonsignori I., Demarchi I., Monzani A., Bona G. (2012). Lipid Profile and Nutritional Intake in Children and Adolescents with Type 1 Diabetes Improve after a Structured Dietician Training to a Mediterranean-Style Diet. J. Endocrinol. Investig..

[14] Chiang J.L., Maahs D.M., Garvey K.C., Hood K.K., Laffel L.M., Weinzimer S.A., Wolfsdorf J.I., Schatz D. (2018). Type 1 Diabetes in Children and Adolescents: A Position Statement by the American Diabetes Association. Diabetes Care.

[15] Potter J.D. (2002). Book Review Eat, Drink, and Be Healthy: The Harvard Medical School Guide to Healthy Eating by Walter C. Willett. 299 Pp. New York, Simon & Schuster, 2001. $25. 0-684-86337-5. New Engl. J. Med..

[16] Shai I., Rosner B.A., Shahar D.R., Vardi H., Azrad A.B., Kanfi A., Schwarzfuchs D., Fraser D. (2005). Dietary Evaluation and Attenuation of Relative Risk: Multiple Comparisons between Blood and Urinary Biomarkers, Food Frequency, and 24-Hour Recall Questionnaires: The DEARR Study. J. Nutr..

[17] Ministry of Health Public Health Services Nutrition Division (2015). Tzameret-Israeli National Nutrient Database 2015.

[18] Abu-Saad K., Endevelt R., Goldsmith R., Shimony T., Nitsan L., Shahar D.R., Keinan-Boker L., Ziv A., Kalter-Leibovici O. (2019). Adaptation and Predictive Utility of a Mediterranean Diet Screener Score. Clin. Nutr..

[19] Galilea-Zabalza I., Buil-Cosiales P., Salas-Salvadó J., Toledo E., Ortega-Azorín C., Díez-Espino J., Vázquez-Ruiz Z., Zomeño M.D., Vioque J., Martínez J.A. (2018). Mediterranean Diet and Quality of Life: Baseline Cross-Sectional Analysis of the PREDIMED-PLUS Trial. PLoS ONE.

[20] Baker-Smith C.M., Flinn S.K., Flynn J.T., Kaelber D.C., Blowey D., Carroll A.E., Daniels S.R., de Ferranti S.D., Dionne J.M., Falkner B. (2018). Diagnosis, Evaluation, and Management of High Blood Pressure in Children and Adolescents. Pediatrics.

[21] Vanderwall C., Eickhoff J., Randall Clark R., Carrel A.L. (2018). BMI Z-Score in Obese Children Is a Poor Predictor of Adiposity Changes over Time. BMC Pediatr..

[22] Cohen J. (2013). Statistical Power Analysis for the Behavioral Sciences.

[23] Agregán R., Popova T., López-Pedrouso M., Cantalapiedra J., Lorenzo J.M., Franco D. (2022). Fatty Acids. Food Lipids.

[24] Mayneris-Perxachs J., Sala-Vila A., Chisaguano M., Castellote A.I., Estruch R., Covas M.I., Fitó M., Salas-Salvadó J., Martínez-González M.A., Lamuela-Raventós R. (2014). Effects of 1-Year Intervention with a Mediterranean Diet on Plasma Fatty Acid Composition and Metabolic Syndrome in a Population at High Cardiovascular Risk. PLoS ONE.

[25] Levran N., Levek N., Sher B., Gruber N., Afek A., Monsonego-Ornan E., Pinhas-Hamiel O. (2023). The Impact of a Low-Carbohydrate Diet on Micronutrient Intake and Status in Adolescents with Type 1 Diabetes. Nutrients.

[26] Nitta A., Imai S., Kajiayama S., Matsuda M., Miyawaki T., Matsumoto S., Kajiyama S., Hashimoto Y., Ozasa N., Fukui M. (2022). Impact of Dietitian-Led Nutrition Therapy of Food Order on 5-Year Glycemic Control in Outpatients with Type 2 Diabetes at Primary Care Clinic: Retrospective Cohort Study. Nutrients.

[27] Alman A.C., Talton J.W., Wadwa R.P., Urbina E.M., Dolan L.M., Daniels S.R., Hamman R.F., D’Agostino R.B., Marcovina S.M., Mayer-Davis E.J. (2014). Cardiovascular Health in Adolescents with Type 1 Diabetes: The SEARCH CVD Study. Pediatr. Diabetes.

[28] Ren Y., Sun S., Su Y., Ying C., Luo H. (2023). Effect of Fruit on Glucose Control in Diabetes Mellitus: A Meta-Analysis of Nineteen Randomized Controlled Trials. Front. Endocrinol..

[29] Ola M.S., Al-Dosari D., Alhomida A.S. (2018). Role of Oxidative Stress in Diabetic Retinopathy and the Beneficial Effects of Flavonoids. Curr. Pharm. Des..

[30] Leyvraz M., Chatelan A., da Costa B.R., Taffé P., Paradis G., Bovet P., Bochud M., Chiolero A. (2018). Sodium Intake and Blood Pressure in Children and Adolescents: A Systematic Review and Meta-Analysis of Experimental and Observational Studies. Int. J. Epidemiol..

[31] Huang L., Trieu K., Yoshimura S., Neal B., Woodward M., Campbell N.R.C., Li Q., Lackland D.T., Leung A.A., Anderson C.A.M. (2020). Effect of Dose and Duration of Reduction in Dietary Sodium on Blood Pressure Levels: Systematic Review and Meta-Analysis of Randomised Trials. BMJ.

[32] Viroli G., Gonçalves C., Pinho O., Silva-Santos T., Padrão P., Moreira P. (2021). High Adherence to Mediterranean Diet Is Not Associated with an Improved Sodium and Potassium Intake. Nutrients.

[33] Vasara E., Marakis G., Breda J., Skepastianos P., Hassapidou M., Kafatos A., Rodopaios N., Koulouri A.A., Cappuccio F.P. (2017). Sodium and Potassium Intake in Healthy Adults in Thessaloniki Greater Metropolitan Area-The Salt Intake in Northern Greece (SING) Study. Nutrients.

[34] Catherine R., Benjemin C., Robert C., Katherine T., Thomes Z. (2012). Moderen Nutrition in Health Ad Disease.

[35] Wu Y., Lu H., Yang H., Li C., Sang Q., Liu X., Liu Y., Wang Y., Sun Z. (2016). Zinc Stimulates Glucose Consumption by Modulating the Insulin Signaling Pathway in L6 Myotubes: Essential Roles of Akt-GLUT4, GSK3β and MTOR-S6K1. J. Nutr. Biochem..

[36] Little P.J., Bhattacharya R., Moreyra A.E., Korichneva I.L. (2010). Zinc and Cardiovascular Disease. Nutrition.

[37] (1998). Dietary Reference Intakes for Thiamin, Riboflavin, Niacin, Vitamin B6, Folate, Vitamin B12, Pantothenic Acid, Biotin, and Choline.

[38] Eshak E.S., Iso H., Maruyama K., Muraki I., Tamakoshi A. (2018). Associations between Dietary Intakes of Iron, Copper and Zinc with Risk of Type 2 Diabetes Mellitus: A Large Population-Based Prospective Cohort Study. Clin. Nutr..

[39] Squitti R., Negrouk V., Perera M., Llabre M.M., Ricordi C., Rongioletti M.C.A., Mendez A.J. (2019). Serum Copper Profile in Patients with Type 1 Diabetes in Comparison to Other Metals. J. Trace Elem. Med. Biol..

[40] Wolff S.P., Dean R.T. (1987). Glucose Autoxidation and Protein Modification. The Potential Role of ‘Autoxidative Glycosylation’ in Diabetes. Biochem. J..

[41] Oost L.J., van Heck J.I.P., Tack C.J., de Baaij J.H.F. (2022). The Association between Hypomagnesemia and Poor Glycaemic Control in Type 1 Diabetes Is Limited to Insulin Resistant Individuals. Sci. Rep..

[42] Pérez-Segura P., de Dios O., Herrero L., Vales-Villamarín C., Aragón-Gómez I., Gavela-Pérez T., Garcés C., Soriano-Guillén L. (2020). Children with Type 1 Diabetes Have Elevated High-Sensitivity C-Reactive Protein Compared with a Control Group. BMJ Open Diabetes Res. Care.

[43] Grandjean A.C. (2012). Dietary Intake Data Collection: Challenges and Limitations. Nutr. Rev..

