# Supplementary material for: The Mediterranean Diet for Adolescents with Type 1 Diabetes: A Prospective Interventional Study
Source: Nutrients. 2023 Oct 27;15(21):4577. doi: 10.3390/nu15214577 (PMC10648751; doi:10.3390/nu15214577)
Supplement: Supplementary file 1 [file nutrients-15-04577-s001.zip › nutrients-2660019-supplementary.pdf]

Supplementary Materials

This Supplementary S1 has been provided by the authors to give readers additional information about their work. Supplement to: Neriya Levran, Orit Pinhas-Hamiel,et al.

Supplementary S1:

Cooking workshop

The workshop's objectives were to explain the benefits of a Mediterranean diet and to promote skills in assembling and preparing Mediterranean dishes.

The syllabus of the workshop - 8 hours

Part 1: A lecture was given by a dietician regarding the aims of the study, the rationale of Mediterranean diet and its apparent advantages, insulin administration, and general information regarding preferred types of foods and daily diet managing.

Part 2: Practical section. The participants prepared about 13 different dishes and became familiar with the main food products that are used in a Mediterranean diet cuisine. During this phase, the participants consumed the foods they prepared and administered insulin, with the guidance of a dietician.

At the end of the cooking workshop, each participant received a booklet with recipes s according to the Mediterranean diet, a shopping list and a detailed weekly menu that lists the amount of carbohydrates for particular meals and foods.

Individual meetings with the dietitian

Each participant met individually, for a 60-minute session, with the dietician in weeks 1,2,4,7,10,12, and 24 of the intervention. Dietary education sessions started with weight and waist circumference measures. After blood pressure and ketone values were measured, the dietician uploaded the glycemic, insulin and carbohydrate data from the pump, CGM and food manager applications (24-hour recall). The dietician asked for feedback, led a discussion on problem-solving, and set clear objectives based on glycemic control and upcoming events (holidays, etc.). At the end of each meeting, a detailed menu was distributed, with recipes and more examples of daily menus.

A WhatsApp group was managed by the dietician, who added diet recipes, notes (prior to holidays) and notifications about upcoming measurements and electronic questionnaires, and reminders for visits.

At the end of the study, after the data analysis, the participants were provided all of their individual data and calculations of trends and normal range values of the nutritional and health parameters that were assessed during the 24-week intervention.

The Mediterranean diet

The Mediterranean diet was prescribed a moderate-fat diet, rich in vegetables and low in red meat, with poultry and fish preferred to beef and lamb. The primary sources of added fat were 30 to 45 g of olive oil and/or a handful of nuts (five to seven nuts <20g) per day.

Below are examples of a two-day menu:

	Day 1	Carbohydrate (gram)	Day 2	Carbohydrate (gram)
Breakfast	2 slices of whole wheat bread+ 2 table spoon of white cheese 5%+pinch of Zaatar+ 6 olives+ 1 sliced cucumber	**37**	Greek yogurt 7% fat+ 7 Walnuts+ Table spoon of blueberry's+ table spoon of Flaxseed	**10**
Lunch	Seasoned chicken breast with 1 cup of rice (146 g) cauliflower (100 g)	**50**	A stew of orange lentils and rice* (320 g) + 2 Chicken skewers 3-4 chicken patty (40 gr each)	**50**
Dinner	Greek salad*+ 2 slices of whole wheat bread+ 1 table spoon (15 g) of Tichina	**45**	2 slices of whole wheat bread+ 1 tomato+ 1 cucumber+1 table spoon of olive oil+1 omelet (1 egg)	**40**

Be sure to add 2 tablespoons of olive oil per day or nuts 30 g 5 times a week

Snacks:

Between the three main meals, two small meals can be eaten during the day.
The following list contains ideas for snacks, and the number of carbohydrates of the items.
A slice of whole bread with peanut butter or almond spread or hummus or lentil spread.
2 Rice crackers with peanut butter.
1 apple
Chocolate 100 g
Examples of items for a shopping list.
Orange, mash, green, black lentils, chickpeas, dry beans in different colors.
Almonds, seeds (sunflower, pumpkin), peanuts, nuts (walnuts, hazelnuts, pistachios) - without salt and not roasted. Let them salt and lighten at home
Whole rice - round for those who like it crunchy or long in my name
Quinoa

Below are the main topics that we focused on during the eight sessions.

<u>Session 1:</u>
Adjusting insulin and the menu more precisely to participants' preferences, and in accordance with the research guidelines

Session 2:
Special concerns, according to the patients' requests: physical activity, bowel movements, hypoglycemia, etc.

Session 3:
Maintaining a Mediterranean diet when eating out, and at family and social events, holidays, and parties.

Session 4:
Healthy eating and eating habits, including avoidance of processed food, avoidance of screen time while eating, and family meals.

Session 5:
Lipids and fats, their proportions in various food items, and the effect on metabolic health

Session 6: Motivational session

Session 7: Conclusion

Each participant had the option to contact the caregiver and dietician any time during the intervention.
Two motivational phone reach outs were conducted by the dietician.
The participants continued regular visits with their endocrinologists.
